# Israel’s oral health policy in reflection of the WHO Global Oral Health Action Plan (2023–2030)

**DOI:** 10.1186/s13584-026-00779-8

**Published:** 2026-09-14

**Authors:** Dara Schwartz, Hagit Domb Herman, Shiri Waingrod, Lena Natapov

**Affiliations:** Division of Dental Health, Ministry of Health, Jerusalem, Israel

**Keywords:** Oral Health Action Plan, Bangkok declaration, Universal health coverage, Oral health, Public health policy

## Abstract

**Background:**

Oral diseases are among the most prevalent non-communicable conditions worldwide, contributing to significant health burdens and disparities. In response, the World Health Organization established the Global Oral Health Action Plan 2023–2030, urging countries to integrate oral health into universal health coverage and adopt comprehensive prevention strategies. This article examines Israel’s alignment with these objectives.

**Main Body:**

Over the past 15 years, Israel has advanced oral health within Universal Health Coverage by integrating dental services into the National Health Insurance Law and progressively expanding public financing to children and the elderly, while maintaining coverage for special patient groups. Children and the elderly now account for over 40% of those eligible for publicly funded dental care. Prevention is central to policy. National school and mother-and-child health programs provide supervised toothbrushing, fluoride varnish applications, and oral health education, reaching nearly all children in recognized educational settings. Multidisciplinary primary care models link dental professionals with nurses and pediatric providers to deliver coordinated, life-course services, while strengthening the oral health workforce. Additional system measures include front-of-pack sugar warning labels, a national policy to phase down dental amalgam use, and ongoing efforts to establish a unified digital surveillance system for oral health. Together, expanded coverage, prevention-focused delivery, workforce development, and system strengthening position oral health as an integral component of Universal Health Coverage in Israel.

**Conclusion:**

Israel has made considerable progress in aligning with the Global Oral Health Action Plan, particularly by expanding coverage and preventive interventions for children and the elderly. However, continued efforts are needed to address remaining gaps in adult coverage, strengthen the capacity and distribution of the oral health workforce, and further integrate oral health care into innovative, multidisciplinary models. Systematic surveillance systems are also needed to accurately track disease burden and ensure equitable outcomes across all population groups.

## Introduction

Oral health is a fundamental part of overall health, influencing well-being and quality of life. Maintaining oral health is essential not only for preventing oral diseases but also for reducing the risk of chronic health conditions. Despite being largely preventable, these conditions remain among the most prevalent chronic conditions globally, affecting more than 3.5 billion people [1]. Recognizing this substantial global burden, the World Health Organization (WHO) adopted the Global Oral Health Action Plan (GOHAP) 2023–2030 [2], a comprehensive initiative aimed at addressing these issues through coordinated global efforts.

Israel, despite its comprehensive health system, has become a strong advocate for integrating oral health within the global health agenda. While the country operates under the National Health Insurance Law (NHIL), which guarantees universal access to a defined basket of health services, dental care has historically been excluded from the publicly financed benefits package and has operated largely in a privately financed, out-of-pocket market [3]. This structural separation created a fundamental inconsistency within an otherwise universal system and contributed to a market failure as access to oral health services depended primarily on individual financial capacity rather than population need.

However, significant changes have been made in recent years. A key milestone was the introduction of the dental care reform in 2010, which expanded dental coverage to children and later to the elderly, substantially broadening entitlements under the NHIL [4, 5]. While these reforms transformed the financing architecture of oral healthcare, expanded coverage has not fully translated into equitable service utilization, and some disparities still persist across socioeconomic and geographic groups [5, 6].

Israel has actively promoted the GOHAP through international engagement. In February 2020, Israel was among 17 countries that co-supported a landmark statement at the 146th session of the WHO Executive Board, calling for the integration of oral health into Universal Health Coverage (UHC) [7]. This engagement contributed to the subsequent adoption of the 2021 World Health Assembly Oral Health Resolution and informed the development of the GOHAP 2023–2030. Israel’s involvement continued with its co-sponsorship of a side event at the 2023 WHO High-Level Meeting on UHC, which focused on integrating oral health into national health systems and reinforcing commitment to equitable access through UHC.

In 2024, Israel reaffirmed its commitment to the GOHAP by submitting a set of national pledges at the Global Oral Health Conference in Bangkok. Israel has expressed its intention to explore opportunities to broaden age-based dental care as part of UHC, with particular attention to extending coverage to more elderly individuals and enhancing access to preventive dental treatments for the adult population. Other objectives include addressing accessibility issues in remote and underserved geographic locations, with a focus on reducing wait times for specialist dental treatment. These pledges reflect the continuation of Israel’s efforts to align national oral health policies with the GOHAP objectives and form a basis for further assessment of existing gaps.

The goal of this article is to analyze Israel’s policy directions within the key domains of the WHO’s GOHAP (2023–2030) [2]. By examining Israel’s strategies, the article explores how the country seeks to strengthen the integration of oral health into broader health systems, promote equitable access to care, and enhance long-term health outcomes. In doing so, we will review Israel’s oral health policy through the lens of the GOHAP, assessing the extent to which its goals have been realized.

### Global Oral Health Action Plan (2023–2030)

The GOHAP (2023–2030) provides a structured policy framework for evaluating national oral health systems. It defines two overarching goals [2]: integration of oral health into UHC and [2] reduction of the oral disease burden, operationalized through six strategic objectives and measurable targets [2]. These domains serve as the analytic structure for assessing Israel’s policy alignment. Table 1 summarizes Israel’s progress toward these targets as of 2024. While substantial advances have been achieved, universal entitlement across the full adult life course has not yet been achieved.

**Table 1:** Overarching Global Targets and Strategic Targets for Oral Health Improvement by 2030

Target Area	2030 Global Target	Israel Status (as of 2024)	Metric / Evidence
Overarching Global Targets			
UHC for Oral Health	By 2030, 80% of the global population are entitled to essential oral health services	Partially Achieved	Coverage for children, elderly, and special populations; adults 19–71 excluded.
Oral Disease Burden Reduction	By 2030, the combined global prevalence of the main oral diseases and conditions over the life course shows a relative reduction of 10%	Partially Achieved	Periodic surveys and HMO data provide trend information; national surveillance still developing with improved measurement expected by 2030.
Strategic Targets by 2030			
1. Oral Health Governance	1.1 National policy + dedicated MoH staff	Achieved	Oral health policy embedded in NHIL; managed by Division of Dental Health.
	1.2 Phase-down of dental amalgam use	Achieved	Amalgam restricted for children < 15, pregnant and breastfeeding women; composites subsidized.
2. Health Promotion & Disease Prevention	2.1 Policies to reduce free sugar intake	Partially Achieved	Red labeling and healthy lifestyle initiatives; sugar tax introduced in 2022 and repealed in 2023.
	2.2 National guidance on fluoride delivery	Partially Achieved	Fluoride guidelines exist; community water fluoridation discontinued.
3. Health Workforce	3.1 Workforce policy + training	Achieved	Two dental schools, specialist training, and licensing exams. A national workforce strategy focusing on expansion of domestic training capacity.
4. Oral Health Care	4.1 Integration into primary health care	Achieved	“Tipat Halav” clinics and school-based prevention programs.
	4.2 WHO dental preparations in essential medicines list	Achieved	Essential dental medicines and preventive agents available and subsidized for eligible populations.
5. Oral Health Information Systems	5.1 Monitoring frameworks	Partially Achieved	High digital integration within oral health care system, but a unified national monitoring is currently lacking.
6. Oral Health Research Agendas	6.1 National research agenda for public health interventions	Partially Achieved	Institutional research and periodic surveys are ongoing; coordinated national epidemiological monitoring is under development.

### Oral health governance, universal health coverage and oral health care (aligned with goals 1, 4)

Israel has an advanced healthcare system that ranks high on global health indicators, such as life expectancy, infant mortality, and overall health outcomes [8]. It is anchored in the NHIL where the health system integrates and emphasizes efficient resource use, preventive care, and early disease detection [9]. A sophisticated digital infrastructure supports these efforts, including the widespread adoption of electronic health records (EHRs) [10], telemedicine services [11], and the use of advanced medical technologies such as artificial intelligence in diagnostics and digital health monitoring.

Over the past 15 years, Israel has also made significant progress in expanding access to oral healthcare. Prior to 2010, most dental services in Israel were financed out-of-pocket through the private sector, with limited hospital-based coverage for oral and maxillofacial care. A major reform in 2010 amended the NHIL to introduce universal dental coverage for children, initially up to age 8 [4]. Eligibility was gradually expanded to include preventive and restorative treatments for all children up to age 18 by 2019 [4]. In the same year, publicly funded dental services were extended to adults aged 75 and above, including preventive and restorative care, with prosthetic treatments added for those aged 80 and above [5]. In 2022, eligibility was further lowered to age 72 for both restorative and prosthetic services [5]. To improve access for institutionalized elderly populations, mobile dental units were introduced to provide on-site care in nursing homes with 150 or more residents. Collectively, these reforms established a publicly financed framework in which essential preventive services are provided free of charge and other treatments are offered with minimal subsidized copayments.

Beyond age coverage, the NHIL includes a specialized basket of services for individuals from special population groups. This encompasses comprehensive care for individuals with congenital syndromes and neurodevelopmental disorders. It also provides a “safety net” for oncology patients by covering essential oral treatments required before, during, and after cancer therapy. Ongoing expansions of the basket aim to broaden these services and strengthen the continuity of care.

Despite these improvements, many adults aged 19 to 71 who are not covered by publicly funded programs continue to rely on supplemental private insurance offered by Health Maintenance Organizations (HMOs). While this supplementary insurance (Shaban), used by approximately 81.5% of the population, is generally considered affordable, it may still present access barriers for some individuals [12].

Achievement levels of public dental coverage in Israel (the proportion of the total population [13] covered by the public dental program) expanded gradually beginning in 2010 and reached 41% by 2022. In 2010, coverage started at 18%, targeting children aged 0–8 years. This achievement level rose steadily each year, reflecting successive expansions of the eligible age groups for dental services: 21% in 2011 (ages 0–10), 25% in 2012 (ages 0–12), and 31% in 2018 (ages 0–16).

By 2019, with full inclusion of children aged 0–18 years, the achievement level for this group reached 34%. In the same year, the gradual expansion of universal oral health coverage was extended to the elderly, beginning with the inclusion of individuals aged 75 and above and 80 and above, corresponding to achievement levels of 2% and 3%, respectively. Further reforms expanded elderly coverage to include those aged 72 and above, raising their achievement level to 7% in 2022. Throughout this period, coverage for minors remained stable at around 34%. The combined coverage of children and the elderly increased from 39% in 2019 to 41% in 2022. (Table 2).

**Table 2:** Universal dental coverage in Israel: service uptake by age group and year (2009–2022)

Year	Age group	Population covered (*N*)	Total population (*N*)	Achievement level	Policy change
2009	NA	NA	7,485,600	NA	Child Dental Care Reform Planning
2010	0–8	1,347,900	7,623,600	18%	Start of the Child Dental Care Reform
2011	0–10	1,654,200	7,765,800	21%	Age expansion
2012	0–12	1,964,000	7,910,500	25%	Age expansion
2016	0–14	2,415,600	8,546,000	28%	Age expansion
2017	0–15	2,604,100	8,713,300	30%	Age expansion
2018	0–16	2,794,600	8,882,800	31%	Age expansion
2019	0–18 75–80 80+	3,560,200 164,800 269,700	9,054,000	34% 2% 3% **Total: 39%**	Full coverage of children Elderly dental care reform: 1. 75 + preventive restorative 2. 80 + preventive, restorative, prosthodontic
2022	0–18 72+	3,266,700 680,500	9,557,500	34% 7% **Total: 41%**	Reform expanded to 72+ (preventive, restorative, prosthodontic treatment) Mobile dental care for institutionalized elderly (150 + residents)

### Oral health promotion and disease prevention (Aligned with Goals 2.1 & 2.2)

#### Oral disease burden in children

Oral disease remains one of the most prevalent chronic health conditions among children in Israel, with dental caries affecting a substantial proportion of the population. National surveys conducted before the reform revealed high levels of untreated decay and low rates of caries-free children, highlighting longstanding gaps in prevention and care.

Among five-year-old children, national surveys conducted in 1989 and 2007 reported average dmft scores of 2.72 and 3.31, respectively [14, 15]. In 2007, 2.71 of the teeth were untreated decayed (dt), and only 0.49 were filled (ft) [15]. The proportion of caries-free children declined from 41% in 1989 to just 35% in 2007 [14, 15]. The implementation of the dental reform, initiated in 2010 and gradually expanded to include all children up to age 18 by 2019, led to improvements in oral health outcomes. By 2016, the average dmft among six-year-olds decreased to 2.56, with a measurable increase in the number of filled teeth (ft), reaching 1.15 [16].

Similarly, among 12-year-olds, the average DMFT decreased from 2.99 in 1989 [14] to 1.66 in 2002 [17], while the caries-free rate improved from 21%(12) to 46% [17] over the ame period. In 2002, the (DT) component was 0.91, and the (FT) was 0.72 [17]. In 2023, after the implementation of the reform, national data showed that the average DMFT among 12-year-olds slightly declined, with an average of 1.37, DT of 0.64, FT of 0.72, and 51% of children bing caries-free [18].

#### Oral disease burden in the elderly

Oral disease among the elderly remains a significant public health concern in Israel. Limited public coverage and high out-of-pocket costs created substantial barriers to dental care for this population. In 2004, 54.4% of community-dwelling adults aged 65 + were completely edentulous [19]. Additional findings from the “Mabat Zahav” national health and nutrition survey showed that only 45% of adults in this age group rated their oral health as good or very good, and just 38.6% had visited a dentist in the previous six months [20]. Many reported functional limitations related to oral health, including pain, difficulty chewing, and reduced social participation, captured by OHIP (Oral Health Impact Profile) indicators.

However, in recent years, oral health among the elderly has shown measurable improvement coinciding with the gradual expansion of publicly funded dental care. A recent study found that the proportion of completely edentulous adults aged 65 and above had declined to approximately 24%, while 10% retained all their natural teeth [5]. Among those aged 65–74, 18.5% were edentulous, and dentate individuals had an average of 20.3 teeth. In contrast, among those aged 85 and above, 38% were edentulous, with an average of 13 remaining teeth [5].

#### Periodontal disease surveillance and trends

National surveillance of periodontal disease in Israel is currently limited by the absence of a routine monitoring framework. Available evidence is therefore derived primarily from record-based studies rather than systematic national surveys. Recent evaluations of more than 57,000 military personnel aged 18–50 indicate a periodontitis prevalence of approximately 9.79% [21, 22]. A clear social gradient is also evident, with lower socioeconomic status and lower intellectual capability scores as significant predictors of greater need for invasive periodontal treatment [23].

#### Oral cancer surveillance and trends

National surveillance of lip, oral cavity, and pharyngeal cancers in Israel provides insight into the country’s oral health challenges. While the Israel National Cancer Registry (INCR) remains the primary source for monitoring, recent analysis reveals a stable but persistent burden. According to data through 2024–2025, these malignancies account for approximately 2.3%-3.0% of all new invasive cancer cases [24].

The primary modifiable risk factors for oral cancer in Israel are tobacco smoking and alcohol consumption. National burden-of-disease modeling identifies smoking as the dominant preventable carcinogenic exposure, accounting for approximately 21% of all invasive cancer cases, while alcohol contributes a smaller but measurable 1.5% [25]. Despite the continued relevance of tobacco control as a core prevention strategy, smoking rates have plateaued at approximately 20.9% among adults [26].

#### Oral health promotion and prevention programs

Recognizing the close association between oral health and non-communicable diseases (NCDs), Israel has placed increasing emphasis on prevention and health promotion in community settings. This approach reflects growing recognition of shared behavioral risk factors, particularly high sugar consumption, unhealthy diet, and inadequate hygiene practices. Accordingly, oral health has been integrated into broader national public health strategies, including the Efsharibari program (Israel’s National Program for Healthy Living) [27], which promotes healthier lifestyles through improved nutrition, increased physical activity, and disease prevention.

Within this national strategy, the branded initiative “Smile Efsharibari” serves as the dedicated oral health promotion framework. The program focuses on reducing sugar intake, encouraging routine oral hygiene practices, and strengthening structured health education in kindergartens and schools. Practical components include supervised toothbrushing with fluoride toothpaste and the application of fluoride varnish, reinforcing preventive behaviors early in life.

In alignment with the GOHAP’s strategy, the Ministry of Health (MOH) also funds several child-focused, community-based oral health programs under the NHIL, designed to ensure equitable access to preventive care. These include the School Dental Services (SDS) [4] and the Oral Health and Caries Prevention Program delivered through the Mother and Child Healthcare Centers (“Tipot Halav”) [28], supporting a life-course and community-oriented prevention strategy.

#### A. School dental services

The School Dental Service (SDS) is a key component of Israel’s public oral health system for children. Initially operated by local authorities with joint funding from the MOH and nominal parental contributions, the SDS provided preventive and basic restorative care to school-aged children before their integration into the national health system. In 2010, the SDS became universal and fully funded under the NHIL, offering primary, community-based oral health services for kindergarten and schoolchildren. Today, the SDS is provided mostly by the Federation of Local Authorities’ service providers.

The SDS offers a wide range of preventive activities, including oral health education, distribution of toothbrushes and toothpaste, annual dental check-ups, and referrals for treatment. The “Shenli” oral health promotion program is culturally and linguistically adapted to Israel’s diverse populations and is implemented nationwide in kindergartens and schools. The program provides age-appropriate education and equips children with practical skills to maintain daily oral hygiene. In addition, it promotes healthier lifestyle behaviors, including reduced sugar consumption and preventive health awareness from an early age. Additionally, a supervised toothbrushing and fluoride application program operates in kindergartens for children aged 3–5, with a specific focus on underserved communities. A fluoride varnish program was recently introduced in primary schools in socioeconomically disadvantaged areas as a pilot initiative to reduce caries rates, with plans for possible future expansion.

As of the 2024–2025 school year, the SDS program provides nationwide coverage for nearly 2 million children aged 3 to 15 (from nursery through eighth grade). Among preschoolers aged 3 to 5 in state-funded preschools, approximately 535,500 children [29] are eligible for supervised toothbrushing, with additional fluoride varnish programs implemented in low socioeconomic areas. For school-aged children between 6 and 14 years (those in the compulsory education system), about 1.5 million are eligible for preventive dental services through the SDS, resulting in an estimated 96% coverage rate. Overall, the program is close to achieving universal coverage through community-based oral health prevention initiatives for the age groups mentioned (Table 3).

**Table 3:** School dental service entitled and achievement levels- estimated data*

Educational Institution	Age group	Eligible Population (*N*)	Total population (*N*)	Achievement Level (%)
Kindergarten	3–5	535,000	554,500	96%
School	6–14	1,509,000	1,570,200	96%
Total		2,044,000	2,124,700	96%

*Data sources:

Ministry of Education, 2024–2025-year school data- Eligible Population [29]

Israeli Central Bureau of Statistics 2023- Total Population [13]

#### B. Mother and child healthcare centers (“Tipot Halav”)

Mother and Child Healthcare Centers (MCHCs) play a central role in Israel’s public health system. Fully funded by the MOH, they provide comprehensive preventive services to mothers and children from birth through six years of age. Their activities emphasize early prevention, parental guidance, and health education, thereby supporting healthy development during the critical early-life period.

In 2017, the MCHC Oral Health and Caries Prevention Program, was launched as a pilot initiative and has since steadily expanded. Over the past year, the program has grown significantly, with plans to implement it across all MCHCs. Between 2018 and 2025, the MCHC Oral Health and Caries Prevention Program was implemented in approximately 100 out of 447 MCHCs operated by the MOH, representing 22% of MOH-run settings.

Initially implemented by public health nurses, the program was expanded in 2024 to additional centers through a multidisciplinary workforce model that includes dental hygienists and dentists. The program aims to promote proper oral hygiene practices, including daily toothbrushing with fluoride toothpaste and healthy eating habits to reduce sugar consumption and prevent dental caries. It also provides preventive treatments, such as professional fluoride applications, and employs a caries risk assessment tool and a preventive treatment protocol to guide care [28]. When children require more complex dental care, MCHCs refer them to HMO dental clinics to ensure appropriate follow-up and effective management of their ongoing oral health needs.

#### C. Oral health prevention in daycare centers- pilot program

To extend the reach of early childhood oral health prevention beyond MCHCs, a pilot oral health program has been introduced in daycare centers. The pilot incorporates core components of the MCHC Oral Health and Caries Prevention Program, including oral health education, promotion of daily toothbrushing with fluoride toothpaste, and early identification of caries risk. Integrating preventive oral health activities into daycare environments enables earlier exposure to healthy behaviors and provides a basis for evaluating future expansion to additional early childhood frameworks.

#### Regulatory and fiscal policies for sugar reduction

Israel introduced regulatory measures to reduce free sugar consumption through food labeling reforms. In 2010, the Public Health (Food) Law was amended to require nutritional labeling on pre-packaged foods, including the declaration of sugar content [30]. This was followed by the “Efsharibari” (Healthy Choice) reform, which implemented front-of-pack red warning labels for foods and beverages high in sugar, sodium, or saturated fat, effective January 2020. Evaluation of this labeling policy in Israel showed that within three years of implementation, 60% of households reported preferring unlabeled alternatives within unhealthy categories, and approximately 50% of respondents reported purchasing fewer red-labeled products [31].

Recognizing that informational labeling alone may be insufficient to curb consumption among high-risk groups, policymakers shifted toward more direct fiscal interventions. A tax on sugar-sweetened beverages (SSBs) was implemented in Israel in January 2022, and was repealed in March 2023. When the tax was in effect, there was a ~ 5% reduction in per-capita purchases of taxed beverages overall and an approximately 10% reduction in purchases of high-sugar beverages, with larger declines observed in specific population groups [32]. Moreover, sales of large “full-taxed” beverages declined by 24.1%, while large “reduced-tax” beverages (diet/juices) declined even more sharply by 30.7% [33, 34].

#### National policy on optimal fluoride delivery

Israel began implementing water fluoridation in 1981 [35], starting in Jerusalem and gradually expanding to major cities such as Tel Aviv and Haifa. By the early 2000s, approximately 75% of the population had access to fluoridated tap water [35], a period during which Israel also began expanding the use of desalinated seawater as a major drinking water source. Fluoridation contributed to significant improvements in children’s oral health and a marked reduction in dental caries [35]. In 2014, the national fluoridation program was discontinued following a policy change driven by political decisions, despite recommendations from public health experts. In the context of widespread desalination, the cessation of fluoridation resulted in persistently low fluoride concentrations in drinking water. Subsequent studies documented a measurable increase in caries prevalence and treatment needs, particularly among children in lower-income and underserved communities [36, 37].

As noted earlier, the MOH promotes supervised toothbrushing and fluoride varnish programs as part of an integrated fluoride strategy. Community-based and individually delivered interventions operate alongside systemic approaches, reflecting a complementary model of prevention. Given Israel’s predominantly desalinated water supply and low baseline fluoride concentrations, these measures strengthen preventive protection across population groups.

In 2021, national fluoride guidelines were revised, including the withdrawal of recommendations for low-fluoride toothpaste for young children, and updated guidance was issued. The revised guidelines recommend the use of toothpaste containing 1,000 ppm fluoride from the eruption of the first tooth until age six, alongside professionally administered topical fluoride treatments [38]. These recommendations are particularly relevant in a setting where desalinated drinking water provides little baseline fluoride exposure.

### Amalgam policy (aligned with goal 1.2)

The GOHAP also encourages accelerating the phase-down of dental amalgam use, in accordance with the Minamata Convention on Mercury, due to the environmental risks associated with mercury-containing materials, particularly during their use and disposal. In 2018, Israel aligned its national policy with the European Union Directive, recommending reduced use of dental amalgam and limiting removal or replacement to situations of clinical necessity [39]. Prior to this, the use of amalgam had already been restricted for children under 6 years of age and for pregnant or breastfeeding women, and in 2022, the MOH extended these restrictions to children under 15 [39]. Most recently, Israeli professional dental organizations have reviewed the future role of dental amalgam in light of the World Dental Federation’s (FDI) recommendation to phase out its use by 2034, consistent with the COP6 decision under the Minamata Convention on Mercury [40].

### Oral health workforce policy and oral health care (aligned with goals 3,4)

#### Dentists and dental specialists

Israel’s dental workforce plays a central role in the country’s oral health system and has grown steadily over the past decades. Israel has experienced a significant influx of dentists due to the immigration of highly trained academic professionals. Licensing exams in dentistry were introduced in 1992 to ensure that dental practitioners entering the Israeli healthcare system meet sufficient quality standards [41]. The number of licensed dentists increased from 9,130 in 2008 to approximately 13,679 in 2023, raising the dentist-to-population ratio from 1.20 to 1.39 per 1,000 residents (42). Over the same period, the number of active practitioners grew from about 5,700 to nearly 8,000, resulting in an active dentist ratio of approximately 0.8 to 0.84 per 1,000 residents [42].

Approximately 75% of dentists practicing in Israel were trained abroad, about half in Eastern Europe, while only 25% are graduates of the country’s two domestic dental schools [42]. The proportion of dental specialists has remained relatively stable at around 10% over the past two decades [42]. Most dentists and specialists are concentrated in central urban areas such as Tel Aviv and Jerusalem, where dentist-to-population ratios can reach 1.3 per 1,000 residents [42]. In contrast, peripheral regions like the South have considerably lower coverage, with ratios as low as 0.5 per 1,000 [42].

#### National framework for dental specialization

There are approximately 40 accredited residency programs across nine recognized specialties: Oral and Maxillofacial Surgery, Orthodontics, Pediatric Dentistry, Periodontics, Prosthodontics, Endodontics, Oral Medicine, Oral Pathology, and Dental Public Health [43]. Traditionally, these programs have been centralized within hospital-based departments and the country’s two academic dental schools (Hebrew University-Hadassah and Tel Aviv University).

A structural limitation of the traditional university-based specialization model has been its constrained training capacity, which has affected the growth and geographic distribution of the specialist workforce. In response, the MOH introduced targeted support mechanisms to promote the establishment of residency programs in peripheral regions. These initiatives aim to expand training capacity while strengthening service provision in underserved areas. The MOH Support Criteria (“Mivchanei Tmicha”) [44] framework is aimed at incentivizing the opening of periphery residency programs in northern and southern districts. Current strategic initiatives are now focused on integrating residency programs into HMOs.

#### Dental Hygienists

Dental hygienists play a vital role in the oral health system, emphasizing prevention and patient education, especially in community and public health settings. They are trained through two-year accredited certificate programs, similar to associate degree programs in other countries. These training programs prepare hygienists to deliver care that contributes to wider health promotion and prevention efforts. Candidates must pass a national licensing exam to obtain a professional practice license.

The number of licensed dental hygienists up to age 67 has shown a modest increase over the past decade, from 2,205 in 2015 to 2,752 in 2023, and the ratio per 1,000 people remains relatively low, rising only slightly from 0.26 to 0.28 during that time [42].

#### Collaborative oral health care workforce

Aligned with the WHO’s framework on interprofessional education and collaborative practice, Israel is working to advance integrated care models that embed oral health within the broader healthcare system. These efforts seek to strengthen collaboration between dental professionals and other healthcare providers, such as public health nurses and pediatricians [45], to improve care coordination and expand access to preventive dental services.

A notable example illustrates of this is the MCHC Oral Health and Caries Prevention Program which integrates oral health into the established management duties and daily routines of public health nurses. By incorporating oral health topics into continuing education [46], the system enables nurses to serve as coordinators within the multidisciplinary MCHC environment. This setup enables a synchronized approach in which dental hygienists and nurses work together to ensure that caries risk assessment and preventive guidance are part of the child’s routine health visits.

### Oral health information systems (aligned with goal 5)

The MOH and HMOs have promoted the use of digital tools and invested in infrastructure to support innovation in healthcare delivery. As a result, digital health solutions such as big data analytics, personalized medicine, and telemedicine have become increasingly integrated into the national health system, aligning with the GOHAP’s goals to strengthen health surveillance and information systems.

Digital tools have become foundational to the Israeli healthcare experience, streamlining access through online scheduling and mobile applications that support both general wellness and chronic disease management. While these platforms were already evolving, their application in dentistry was significantly accelerated by recent global health challenges. To maintain continuity of care, the MOH encouraged HMOs and hospitals to expand remote consultation capabilities. These initiatives established a precedent for integrating tele-health into the public framework, providing essential triage and specialist consultation beyond emergency needs [9, 11].

These digital capabilities have proven equally vital during national emergencies, where maintaining access to oral health services remains a logistical challenge. Tele-dentistry platforms now enable remote triage and digital prescription services, significantly reducing strain on physical emergency facilities and minimizing unnecessary travel for patients. Furthermore, national health portals and AI-based diagnostic tools support safer care delivery during crises by providing real-time updates and remote monitoring [47]. These innovations are particularly critical for reaching vulnerable populations, including the elderly, individuals with disabilities, and residents in high-risk areas, ensuring that essential oral healthcare remains accessible regardless of external constraints [9, 47].

Private dental practices have also benefited from rapid digital advancements, including enhanced patient communication systems, appointment management platforms, electronic health records, and digital imaging technologies. Israel’s broader high-technology ecosystem and strong adoption of medical innovation have positioned dentistry among the more digitally advanced health sectors. However, challenges remain [48]. Variability in digital training, resource allocation, and technical infrastructure may affect the pace and extent of implementation across clinics. Consequently, the integration of digital innovations in private practice settings is uneven, affecting their potential to optimize service quality and efficiency.

## Discussion

The GOHAP primarily evaluates national policy commitments, governance structures, and the availability of essential oral health services rather than individual patterns of service utilization or clinical outcomes. Accordingly, alignment with WHO goals reflects the extent to which oral health services are integrated into health systems and made accessible at the population level. At the same time, gaps may arise between policy entitlement and real-world access due to broader social, economic, and geographic factors, and acknowledging these gaps is essential for a balanced assessment of progress under the GOHAP framework.

Over the past 15 years, Israel’s oral health policy has consistently pursued universal coverage for dental services and integrated oral care into the general healthcare system, in line with GOHAP objectives. Major national policy developments, including public funding of dental care for children and for the elderly under the NHIL, reflect a life-course approach to oral health and a strong commitment to equity and access to care [4, 5]. These reforms place Israel among other countries that have incorporated dental services into national health coverage for defined population groups.

From a universal coverage perspective, Israel has made meaningful progress toward the WHO’s 2030 target of ensuring that 80% of the population has access to essential oral health services. Publicly financed dental care currently covers children up to age 18 and the elderly aged 72 and above, together accounting for more than 40% of the total population. While this represents substantial progress relative to the pre-reform period, population-wide coverage has not yet been achieved.

The decision to prioritize children and the elderly was evidence-based and ethically grounded. Early-life dental interventions promote healthier oral health trajectories [49] and reduce long-term treatment needs, while providing dental care supports dignity, quality of life, and the management of chronic conditions [50]. Together, these policies align with international public health principles that emphasize prevention and equity across the life course.

These reforms have yielded measurable benefits, including improved access to care, reduced out-of-pocket costs for families, and strengthened oral health behaviors [5, 6]. Extending publicly funded dental services to additional high-risk groups, such as individuals with disabilities, populations with systemic or at-risk conditions, and residents of long-term care facilities, further reflects Israel’s capacity to address complex health needs. Collectively, these developments indicate substantial progress in expanding the reach of publicly financed oral health services.

Despite these advances, socioeconomic and ethnic disparities in oral health outcomes still persist. Elderly individuals experiencing financial hardship remain significantly more likely to be edentulous, and cost remains a barrier to completing dental treatment, even with expanded coverage [5]. Marked ethnic disparities are also evident, with edentulism more than twice as prevalent among minority population groups than among the Jewish population [5]. These patterns indicate that expanding coverage alone does not ensure equitable outcomes and highlight the continuing influence of broader social determinants of health.

A notable gap remains among the elderly aged 65–71. Although the National Insurance Institute defines “elderly” as men aged 67 and older and women aged 60–65 [51], eligibility for publicly funded dental care begins only at age 72. As a result, only about 60% of adults aged 65 and older are currently covered, leaving a substantial proportion without access to publicly financed dental care. Additionally, a large segment of the working-age population between 19 and 71 remains without such coverage. While supplementary health insurance (Shaban) provides the population with access to a broad range of dental services, these plans remain voluntary and privately financed, requiring additional premiums despite being administered through health maintenance organizations. Reliance on supplementary insurance, therefore, represents a structural limitation in achieving universal oral health coverage as defined by the GOHAP.

While current data limitations prevent a precise quantification of the decline, recent studies suggest that the overall burden of oral disease in Israel has decreased gradually, though improvements remain inconsistent across different population groups. These trends reflect long-term preventive efforts and expanded access to care; however, population-level progress coexists with persistent disparities. Higher levels of dental caries continue to be reported among children from lower socioeconomic backgrounds, where even modest co-payments may limit the effective use of publicly funded services [52]. Similar patterns are observed among the elderly, while tooth retention has improved compared with previous decades, approximately one-quarter of adults aged 65 and above remain edentulous, with prevalence increasing with age and socioeconomic disadvantage.

National oral health epidemiological surveys provide valuable input for planning and evaluation, yet surveillance in Israel remains limited. Data collection relies largely on periodic assessments of children at selected index ages, reflecting historical international monitoring conventions. Regular, comprehensive epidemiological surveillance of adults and elderly populations remains fragmented or outdated. The absence of a systematic, population-wide surveillance framework limits the ability to track trends over time and to evaluate whether expanded coverage translates into improved outcomes among aging populations.

Access to dental services is further shaped by dimensions that are not routinely measured. In Israel, although waiting times are systematically monitored in other areas of healthcare, they are not currently tracked through standardized national metrics for oral health services. While administrative guidance exists for pediatric dental services [53], comparable benchmarks have not been established for the elderly or the general population. Although efforts are underway to develop standardized access measures, the absence of routine monitoring for oral healthcare limits the ability to assess service timeliness and availability across population groups.

Similarly, although disparities in service availability between urban and peripheral regions are well recognized, there is limited systematic national analysis of dental service availability and accessibility. The absence of structured national tools to assess service provision limits understanding of how geographic factors influence real-world access, particularly for vulnerable populations. Although geographic distribution is not an explicit GOHAP target, it remains closely linked to the broader objective of ensuring effective access to essential oral health services.

Preventive care is a particular strength of Israel’s oral health strategy. The SDS oral health promotion and fluoride varnish programs, along with oral health services in MCHCs, collectively embody an early-intervention approach targeting key populations. These efforts are part of a public health-oriented healthcare model and are closely aligned with the targets of the GOHAP.

The SDS program reports a high overall coverage rate, approaching 96% among kindergarten and school-aged children enrolled in recognized educational frameworks. Although all children aged 3–18 in Israel are entitled to compulsory education [54], a 4% discrepancy emerges when comparing data from the Ministry of Education, based on school grade levels, and the Central Bureau of Statistics, based on chronological age. This gap likely reflects differences in population definitions and a mismatch between age and grade level. Furthermore, access to services remains limited for younger children; only those enrolled in publicly funded early childhood education settings are currently covered, leaving those in private preschools outside the program until compulsory preschool begins at age five. Among older children, the gap may be partly explained by homeschooling or attendance in unregulated or unreported educational settings.

While these achievements mark significant progress in preventive oral health, further expansion is needed. Extending the program to include additional early childhood educational settings would improve equity. Similarly, although fluoride varnish is routinely applied in kindergartens, a pilot program in primary schools serving disadvantaged communities remains limited in scope. Scaling up these interventions would enhance the program’s reach and impact.

While the implementation of the MCHC Oral Health and Caries Prevention Program in 22% of settings reflects meaningful progress, its current reach remains limited. Expanding the program to all MCHCs, including those operated by HMOs, is an important step toward improving the accessibility and equity of preventive oral health care for young children across Israel.

The program currently operates under various service delivery models. In some locations, public health nurses provide oral health education and basic preventive interventions as part of routine maternal and child health services. In other settings, dental professionals work alongside medical staff to create an integrated, collaborative care model within MCHCs. Strengthening this multidisciplinary approach could enhance the continuity of care, foster interprofessional collaboration, and build a more cohesive oral health workforce within the public health system.

There is a need to further reorient health services toward a model that not only maximizes universal coverage but also translates it into improved oral health outcomes. This model should place prevention, particularly oral health, at the core of care delivery. Given their central role in Israel’s healthcare system, HMOs should be more actively involved in implementing and monitoring preventive oral health services to ensure consistent access, quality, and impact across the population. This includes integrating oral health quality indicators into their routine performance metrics and quality benchmarks. Such measures would help ensure accountability and better align provider incentives with long-term public health objectives. In parallel, the availability, accessibility, and timeliness of oral health services should be systematically evaluated and improved, particularly concerning reducing waiting times.

Despite these preventive efforts, there is a need for more “upstream” policy interventions, specifically those targeting the commercial determinants of oral health. A prime example is the 2023 repeal of the national tax on SSBs. The subsequent repeal of this tax represents a significant setback for the GOHAP objective of reducing free sugar consumption and highlights the vulnerability of public health gains to political change. Without sustained upstream policies to curb sugar intake, the clinical burden on the public dental system, especially among high-risk groups, will likely remain high, as clinical intervention alone cannot fully mitigate the impact of a food environment that promotes excessive sugar consumption.

The discontinuation of community water fluoridation in 2014 represented a significant shift in Israel’s oral health policy. This decision ended a long-standing, cost-effective public health measure that had contributed to reductions in caries rates [36]. Since then, follow-up assessments, including reports from HMOs, have noted an increase in treatment needs, suggesting a possible impact on population-level preventive care [35, 36]. Given that over 80% of Israel’s drinking water now comes from desalination, reintroducing fluoridation at the point of production within desalination plants is not only technically feasible and cost-effective but also essential to restore this important public health measure and ensure equitable protection against tooth decay. Reinstating water fluoridation, alongside public education and professional engagement, would substantially strengthen Israel’s oral disease prevention strategy and reduce disparities in dental health across the population.

Environmental sustainability constitutes an additional dimension of alignment with global health frameworks. National regulations have already curtailed the use of dental amalgam for designated population groups, reflecting compliance with international environmental commitments. Current efforts focus on maintaining the clinical effectiveness, safety, and accessibility of restorative alternatives, ensuring that environmental objectives are upheld without compromising quality of care or equitable access.

To address longstanding challenges in the dental workforce, the MOH has implemented several coordinated policies over the past three decades. To strengthen the national oral health workforce, additional public dental schools should be established, with priority given to peripheral regions. Expanding domestic training capacity could contribute to a more equitable geographic distribution of dental professionals and reduce reliance on foreign-trained practitioners. In addition, evaluating education programs at foreign dental schools using an approach similar to that for medical schools may be an important step toward improving the quality of dental manpower and training.

Alongside these efforts, Israel has sought to strengthen specialist capacity in high-need fields such as pediatric dentistry, prosthodontics, and oral medicine. Targeted funding schemes for specialty training programs have been introduced to incentivize institutions to open additional residency departments and dental resident positions and to encourage trainees to pursue careers in these underserved specialties. These initiatives reflect a broader commitment to reinforcing the dental workforce and better aligning it with national health priorities.

Attention has also been directed toward the preventive workforce, particularly dental hygienists. They play an important role in preventive care and oral health promotion, particularly in community and public health settings. Yet their numbers remain insufficient relative to population needs, with only a modest increase over the past decade. In line with international trends and the expanding scope of practice [55, 56], there is growing support for transitioning to academic pathways. Israel is now considering developing a university-affiliated bachelor’s program in dental hygiene, which could enhance clinical competencies, strengthen professional recognition, and support greater integration into interdisciplinary care.

Israel’s investment in digital health infrastructure provides a strong foundation for the future integration and monitoring of oral health services. National digital health platforms and telemedicine solutions demonstrated considerable value, however, their adoption in independent dental practices appears to remain uneven. While regulatory requirements mandating digital dental records are not uniformly implemented, the continued use of handwritten documentation in some settings may limit data consistency and pose challenges for comprehensive national monitoring and quality improvement efforts. Overall, Israel’s experience illustrates how substantial progress toward GOHAP objectives can coexist with persistent challenges related to coverage gaps, surveillance limitations, access measurement, the scope of prevention, environmental transition, and the translation of policy into outcomes. These dynamics are not unique to Israel and reflect broader tensions faced by health systems seeking to operationalize universal oral health coverage amid demographic change, expanded service entitlements, and evolving preventive and sustainability priorities.

## Conclusion

Israel has made meaningful progress in aligning its oral health policy with the goals of the WHO GOHAP. The integration of dental care for children, the elderly, and vulnerable populations into national health coverage reflects a clear commitment to equity, prevention, and universal access to care. Preventive programs, workforce development, and regulatory measures further support these actions. To fully achieve the GOHAP objectives, continued efforts are needed to expand coverage, strengthen surveillance, and address gaps in access and workforce distribution. Advancing these priorities will help ensure that oral health is treated as an integral part of overall health and that services are accessible to all segments of the population.

## Data Availability

The datasets used and analyzed during the current study are available from the corresponding author on reasonable request.

## Abbreviations

WHO: World Health Organization
GOHAP: Global Oral Health Action Plan
NHIL: National Health Insurance Law
UHC: Universal Health Coverage
NCD: Non–communicable disease
HER: Electronic Health Record
HMO: Health Maintenance Organization
DMFT/dmft: Decayed, Missing, Filled Teeth/decayed, missing, filled teeth
DT/dt: Decayed Teeth/decayed teeth
FT/ft: Filled Teeth/filled teeth
OHIP: Oral Health Impact Profile
MOH: Ministry of Health
SDS: School Dental Services
MCHC: Mother–Child Healthcare Center
