# Correction: Israel’s oral health policy in reflection of the WHO Global Oral Health Action Plan (2023–2030)

**DOI:** 10.1186/s13584-026-00786-9

**Published:** 2026-10-05

**Authors:** Dara Schwartz, Hagit Domb Herman, Shiri Waingrod, Lena Natapov

**Affiliations:** Division of Dental Health, Ministry of Health, Jerusalem, Israel

**Correction: Israel Journal of Health Policy Research (2026) 15:40**

**https://doi.org/10.1186/s13584-026-00779-8**

The original publication of this article contained an incorrect title, this error was only present in the title and not elsewhere in the article. The incorrect and correct information is shown in this correction article, the original article has been updated.

Incorrect title

Israel’s oral health policy in reflection of the WHO Global Oral Health Action Plan (2020–2023)

Correct title

Israel’s oral health policy in reflection of the WHO Global Oral Health Action Plan (2023–2030)

